# Supplementary material for: The Mistletoe and Breast Cancer (MAB) Study: A UK Mixed-Phase, Pilot, Placebo-Controlled, Double-Blind, Randomised Controlled Trial
Source: Cancers (Basel). 2025 Sep 29;17(19):3169. doi: 10.3390/cancers17193169 (PMC12524002; doi:10.3390/cancers17193169)
Supplement: Supplementary file 1 [file cancers-17-03169-s001.zip › cancers-3841628-supplementary.pdf]

Supplementary Materials S1: Inclusion and exclusion criteria for the MAB study

Inclusion criteria

a) Adults 18 years or over

b) Histologically verified early or locally advanced invasive breast cancer (T1 – 3, N0 – 3, M0) without clinical suspicion/evidence of distant metastases. Routine staging for distant metastases should be according to local practice.

c) Planned adjuvant chemotherapy with or without radiotherapy regime

d) Willing to self-administer or have a nominated person e.g., relative to administer subcutaneous injections

e) Eastern Cooperative Oncology Group (ECOG) performance status 0 or 1

f) All other aspects of management as per local multi-disciplinary team (MDT) decision.

g) No active, uncontrolled infection

h) Randomisation within 12 weeks of surgery

i) Applicable to female participants only: non-pregnant and non-lactating, with no intention of pregnancy during chemotherapy, and prepared to adopt adequate contraceptive measures if pre-menopausal and sexually active. Adequate contraceptive measures according to the Clinical Trial Facilitation Group (CTFG) guidance for women of child-bearing potential (WOCBP)[1] state that WOCBP can be recruited after a negative highly sensitive pregnancy test and assurance of an acceptable, effective method of contraception as a minimum until treatment discontinuation. Additional pregnancy testing should not be required.

j) Applicable to male participants and relevant to trial therapy only, the participant should follow the guidance given by the nurse in relation to chemotherapy: No contraception measures are needed for male subjects with pregnant or non-pregnant women of childbearing potential as the Iscador product and placebo can be classified as having no genotoxicity or demonstrated or suspected human teratogenicity/fetotoxicity at subtherapeutic systemic exposure levels, as per the criteria set in the CTFG recommendations related to contraception and pregnancy testing in clinical trials.[1]

k) No concomitant medical, psychiatric, or geographic problems that might prevent completion of therapy or follow-up.

l) There will be no restrictions on MAB participants being involved in concurrent clinical trial as long as the consultant in charge of their care considers it appropriate and the study protocols do not exclude participation in more than one trial.

Exclusion criteria

a) Additional immunomodulatory therapy - for example: Patients receiving immunotherapy or biological therapy for autoimmune disorders, for diseases such as inflammatory bowel disease, inflammatory arthropathies, multiple sclerosis, etc.
b) Receiving endocrine therapy as a stand-alone treatment
c) Patients with known chronic viral infection such as active Hepatitis B, Hepatitis C or HIV
d) Previous invasive breast cancer or bilateral breast cancer (unless treated with surgery or
radiotherapy >5 years ago).
e) Patients not able or willing to give informed consent
f) Known allergy to mistletoe preparations
g) Previous use of mistletoe, in the last 5 years, or current use of mistletoe
h) Acute inflammatory or pyrexial conditions
i) Chronic granulomatous diseases, active auto-immune diseases
j) Hyperthyroidism with tachycardia.

1. http://www.hma.eu/fileadmin/dateien/Human_Medicines/01-AboutHMA/WorkingGro ups/CTFG/2014_09_HMA_CTFG_Contraception.pdf

Supplementary Materials S2: MAB study therapy regimes

A) Example of typical study therapy & maintenance regime for both ISCADOR® M (Maleus) and ISCADOR® P (Pinus)

Induction phase

Week 1 0.01 mg (1.0ml) x3 = total of 0.03 mg Iscador M or P

Week 2 0.1mg (1.0ml) x3 =total of 0.3 mg of Iscador M or P

Week 3 1mg (1.0ml) x3= total of 3 mg of Iscador M or P

Week 4 10mg (1.0ml) x3= total of 30 mg of Iscador M or P

Week 5 20mg (1.0ml) x3= total of 60 mg of Iscador M or P

Classification of reactions

No or minimal response = no or only marginal local skin reactions, maximally 1 cm in diameter.

Optimal response = local skin reactions, maximum diameter between 1 and 5 cm.

Excessive response = local skin reactions, maximum diameter more than 5cm.

A patient mistletoe medication was increased from the lowest dose to their optimal dose. The optimal dose was defined as a dose to which they experience a sustained local skin reaction, still present 24 hours after the injection. Such a reaction determines the dose they remained on for the rest of trial treatment unless they had a skin reaction of ≥5cm. In cases of skin reactions of ≥5cm, the participants dropped to the dose below (table 1) and this became their optimal dose.

B) Treatment & maintenance regime with placebo

Week 1-5

Physiological saline 0.90% w/v of sodium chloride, (1.0ml) x3

There is unlikely to be a sustained local reaction with the saline placebo and the participants will continue the same physiological saline preparation for the remaining time of the study but essentially the same rule applies as for the mistletoe arm: the participant would continue the same physiological saline preparation in week five and this will be called the maintenance dose.

Supplementary Materials S3: MAB study diary

MAB study doc #12, v2.0 29.09.18

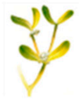

University of BRISTOL

MAB STUDY DIARY: WEEK ☐ TRIAL ID: MAB-☐☐-☐☐☐

	A. Injection details - see note (a)	B. Skin redness and swelling where you injected. Check this **24 hours** after your injection? – see note (b)		C. Any other reactions or comments – see note (c)
		**1. Have you got any redness or swelling?**	**2. If you have a skin reaction, put your plastic circle centrally over it. Use the examples in note (b) to help you answer B2**	**Examples: mood/ energy/ sleep/ feelings/ anything else**
**1**	**Date**: ___/___/___ dd mm yy<br>**Time**: ___ / ___ am / pm<br>hrs mins (please circle)<br>**Dose**: ________ mg	B1. Tick one of the boxes:<br>☐ Yes ☐ No	B2. Is your reaction (tick one of the boxes):<br>☐ Smaller than the circle?<br>☐ Bigger than the circle?<br>☐ As big as the circle at one point or more, but not bigger?	
**2**	**Date**: ___/___/___ dd mm yy<br>**Time**: ___ / ___ am / pm<br>hrs mins (please circle)<br>**Dose**: ________ mg	B1. Tick one of the boxes:<br>☐ Yes ☐ No	B2. Is your reaction (tick one of the boxes):<br>☐ Smaller than the circle?<br>☐ Bigger than the circle?<br>☐ As big as the circle at one point or more, but not bigger?	
**3**	**Date**: ___/___/___ dd mm yy<br>**Time**: ___ / ___ am / pm<br>hrs mins (please circle)<br>**Dose**: ________ mg	B1. Tick one of the boxes:<br>☐ Yes ☐ No	B2. Is your reaction (tick one of the boxes):<br>☐ Smaller than the circle?<br>☐ Bigger than the circle?<br>☐ As big as the circle at one point or more, but not bigger?	

Note: If you are filling in your diary card as a paper version, please bring it with you to each appointment with the nurse or have it by you if you telephone.

Supplementary Materials S4: Participant interview topic guides (A and B)
Interview A
Patients who are participating in the study will be interviewed early in the study therapy phase. The interview will begin with general questions regarding their current health, how their [non-trial] treatment is going generally and how long they have been taking their study therapy. The following topics will then be explored:

Previous knowledge and understanding of mistletoe
- Before you were recruited into this trial had you heard of mistletoe therapy (also called Viscum, Iscador, Helixor etc)?

- If you had heard of it, what do you know about it? Did this match up with the information you have been given for the trial?

- If you hadn't heard of it, what do you think about mistletoe therapy based on the trial information you have been given?

Patients' expectations of the study therapy
- What made you decide to join the MAB trial?
- What do you hope the trial therapy may do for you/help you with?

Acceptability of consenting and randomisation procedures
- How did you feel about being asked to join the mistletoe trial (the process)?
- What did you think about the nurse's approach – the information, signing a consent form?
- It will have been explained by the nurse that you may be randomised to one of two mistletoe therapies or placebo. How do you feel about possibly taking a placebo? - do you think that may change over time?

Experiences and attitudes towards Complementary and Alternative therapies in general and herbal treatments specifically

Mistletoe could be described as a CAM therapy. Talking more generally about CAM
- Have you used any herbal remedies or any other complementary therapies?
- How about family and friends?
- Which other remedies/ therapies? / For what reason? / What or who influenced you to use that? / Did you find it helpful?
- If you have not used CAM remedies/therapies, have you ever thought about them, or about using them?

Quality of life outcome measures & patient experience
The participant will have filled in the questionnaires at the start of the therapy. It is also possible they may have filled in the mid-point questionnaire

- Do the questions ask you about the issues/symptoms/effects that are experiencing or concerning/worrying/ you at this point?
- Are there some issues/ symptoms/ effects that you feel are missed out by the questions?
- Any questions which seemed irrelevant?

Acceptability of questionnaires & patient diary

- How do you feel generally about filling in the questionnaires?

Prompts:
Usefulness: useful/not useful /allows you to self-monitor
Time & effect: too much /about right/ok
Burdensome: difficult/complicated/repetitive
Acceptable: intrusive or inappropriate
Any particular questions those were hard to understand or answer? Or which were particularly helpful (e.g. for self-monitoring)?

Repeat these questions in relation to the patient diary

Adherence to therapy

- The study therapy is taken three times a week by injection – how are you getting on with that? Have you used the 'help' sheets; if so, are they helpful?

(Participant may still be getting nurses help)
Difficult/easy/no real problem
Burdensome
Any practical issues? - injecting, remembering to do it, feeling well enough
Anything which might make it easier/ simpler for you?
Thank you for taking part in this interview.

Interview B

A follow-up interview will be carried out towards the end, or following completion, of the study therapy. Participants who do not complete the study therapy regime will be interviewed shortly after stopping it. The interview will begin with general questions regarding the participant's current health, how their [non-trial] treatment is going generally and how long they have been taking their study therapy/ how long ago they stopped their study therapy. The following topics will then be explored:

Patients' experience of the intervention and its perceived effects – positive and negative

- How have your experiences of the study therapy been?

Acceptability and feasibility of the mode of study therapy delivery

- How did the initial dose adjusting period go for you?
- Did the dispensing (dealing with pharmacy) of the study therapy go ok?

- How did the support you received from the study nurse(s) work?
- The study therapy was to be taken three times/ week by injection – how did you get on with that?

Difficult/easy/no real problem
Burdensome
Any practical issues? (e.g., injecting, remembering to do it, feeling well enough)
Could anything have been done to make it easier/ simpler?

Acceptability of outcome questionnaires
We may have asked you this at beginning of trial but can we ask again

- Do the questions ask you about the issues/symptoms/effects that are experiencing or concerning/worrying/ you at this point?
- Are there some issues/symptoms/effects that you feel are missed out by the questions?
- Any questions which seemed irrelevant? How do you feel generally about filling in the questionnaires?

Prompts:
Usefulness: useful/not useful /allows you to self-monitor
Time & effect: too much /about right/ok
Burdensome: difficult/complicated/repetitive
Acceptable: intrusive or inappropriate
Any particular questions those were hard to understand or answer? Or which were particularly helpful (e.g. for self-monitoring)?

Repeat these questions in relation to the patient diary

Any other treatments or interventions used by the patient

- Have you used any other herbal remedies/complementary therapies or different approaches during the trial period?

Acceptability of participating in the trial

- What are your feelings generally about being involved in the MAB study?

Time
Emotions
Self-care
Health - symptoms
Placebo/ or mistletoe therapy

- Do you think you know whether you were randomised to mistletoe or placebo?
- Why do you think that?

Thank you for taking part in this interview.

Supplementary Materials S5: Healthcare professional interview topic guide

Professional role

- Please could you tell me your job title and give me a brief description of your professional role (length of time in current and other relevant roles)
- Do you have any training in complementary therapies or anthroposophical medicine?

About the Mistletoe study

- What are your views about the likely recruitment (any obstacles to this)?
- How acceptable and feasible do you think the mode of delivery for the intervention will be (sub-cutaneous self-injection taught by a research nurse)?
- What is your view on the likely attrition rate?
- What do you think about the proposed placebo?
- What are your views on the potential for success in blinding?

Views on Mistletoe

We will be using mistletoe as an adjunct treatment to cancer in the MAB trial.

- What do you know about mistletoe as a treatment?

We will be using extract of mistletoe, or 'Viscum album,' as a herbal medicine for people with breast cancer as it may help to ameliorate the side effects of chemotherapy and radiotherapy

- What are your views on this in terms of acceptability to healthcare professionals and patients?

Views on and experience of herbal remedies and complementary therapies

- Have you used herbal remedies or any other complementary therapies?
- How about family and friends?
- Which remedies/therapies? / For what reason? / What or who influenced you/ them to use that? / Did you find it helpful?
- If you have not used CAM remedies/therapies, have you ever considered using them? Why/ not?

Local availability of complementary therapies

- Are you aware of any complementary therapy resources available in your unit / practice / locally?

Role of complementary therapies in cancer

- What role, if any, do you feel complementary therapies might play in cancer care?
- Have any of your cancer patients ever requested information or advice about complementary therapies? How do you respond?

- Do patients ever tell you that they are using complementary therapies? How do you respond?
- Do you ever ask cancer patients whether or not they are using any complementary therapies?
- Have you offered advice or information about complementary therapies to a cancer patient or referred anyone for complementary therapy?
- If so, was that because you thought it would be a good idea or because they had asked?

Complementary therapies and the NHS

- Do you feel complementary therapies should be integrated into the NHS?
- What do you see as the main barriers to integrating complementary therapies into conventional care?
- Are there any organisational / institutional / policy issues that might facilitate or impede such integration?
- Are some complementary therapies, in your view, better candidates for integration than others? Which ones and why?
- Do you think some complementary therapies are harmful? If so, in what way?

Do you have any other comments?

Thank you for taking part in this interview.
